# A survey related to patients’ satisfaction about anaesthetic care at a tertiary care center in Pakistan

**DOI:** 10.12669/pjms.41.11.11811

**Published:** 2025-11

**Authors:** Umair Aftab Baig, Faraz Shafiq

**Affiliations:** 1 Umair Aftab Baig Ex-Resident, Department of Anaesthesiology, Aga Khan University Hospital, Karachi, Sindh, Pakistan; 2 Faraz Shafiq Associate Professor, Department of Anaesthesiology, Aga Khan University Hospital, Karachi, Sindh, Pakistan

**Keywords:** Anaesthesia, Patient Satisfaction, Pakistan, Tertiary Care Centers

## Abstract

**Objective:**

The objective of this study was to assess patients’ satisfaction regarding anaesthetic care at our tertiary care center and to evaluate the common reasons for dissatisfaction.

**Methodology:**

This cross-sectional survey was conducted for a period of seven months from 1^st^ January 2023 to 31^st^ July 2023 at the surgical wards of our tertiary care hospital through a self-completed questionnaire filled by patients themselves. The data was entered and analyzed using SPSS. Frequency and percentages were used to assess the responses and calculate the degree of satisfaction.

**Results:**

The duration of study was seven months included responses from 378 patients. The results were divided into satisfaction regarding preoperative (SA), intraoperative (SB), post operative (SC) care and overall satisfaction (SO). The mean age of patients was 40.6 years. The majority were females who required general surgical procedures, had preoperative workup at anaesthesia clinic and were labeled as ASA-II. Most of them had general anaesthesia. Patients’ satisfaction at SA, SB, and SC was 92.7%, 97%, 94.5% respectively. Two major factors contributed to dissatisfaction were process of taking informed consent (6.4%), and explanation of possible risks and complications (10.4%) associated with anaesthetic care. The commonly reported adverse events in the postoperative period were pain (60%), nausea and vomiting (39.4%), sore throat (24.6%), somnolence (23%), limitations in movement (23.5%) and shivering (15.1%). Amongst them, shivering was the greatest reason for dissatisfaction (14%). Overall, 3.6% of patients were dissatisfied with the anaesthesia care.

**Conclusion:**

Overall, patients’ satisfaction at mentioned intervals was high. The factors associated with dissatisfaction were insufficient counselling about consent, risks, complications and postoperative shivering related to anaesthesia.

## INTRODUCTION

The role of the Anaesthesiologist has always been a hidden aspect of perioperative care. Patients frequently come across their primary physicians during routine visits and usually do not bother to have sufficient knowledge and information about anaesthetic management. Moreover, many of them do not even consider anaesthesiologists as real physicians.^1^ A study conducted in 1999 in Karachi, Pakistan showed poor perception and knowledge among patients about the role of the anaesthesia in perioperative care. Only 56% of participants were aware about the fact that anaesthetists are actual physicians.^2^ Another study conducted in Hyderabad, Pakistan in 2014, showed a similar lack of awareness among study subjects regarding the role of anaesthetists as qualified doctors.^3^ The reasons highlighted in different studies were lack of knowledge, educational background, and limited interaction with respective anaesthesiologist.

A recent study conducted in India revealed that even after having adequate baseline information, the awareness regarding anaesthesia services and role of anaesthetists is not well perceived. This study showed though most respondents (81%) knew that anaesthetists are doctors, nearly half (49.5%) of them considered it as a different specialty. Moreover, only 31% of respondents were satisfied with the counselling provided by anaesthesiologist regarding the procedure, options available and possible complications.^3^ A study conducted in a tertiary health care facility in Ghana revealed that 62.4% respondents had heard about anaesthesia, 85% of which knew that specially trained nurses or doctors are responsible for the delivery of anaesthesia and nearly half of them thought that anaesthesia is all about “putting patients to sleep and waking them up”. Putting all this together, mentioned misunderstandings about anaesthesia affect patient decision making, delayed presentation and poor outcomes.^4^

The aspect of quality assurance in healthcare has received special attention in recent years, including patient satisfaction as one of the key components.^5^ Measuring patients’ satisfaction regarding anaesthetic care is an important concept through which one can improve quality of care and related outcomes.^6^ Patient satisfaction is a complex phenomenon that depends highly on personal judgment.^7^ Adequate information about the procedure and planning contributes to it. This requires subjective analysis of patients’ knowledge, depending upon socioeconomic, psychological and cultural factors, as well as expectations from their anaesthesiologists.^8^ Another important variable in this context is the perception of anaesthesia services. Perceived performance cannot be differentiated from actual performance, when the patients are not familiar with them.^9^ The main objective of this survey was to assess patient satisfaction regarding anaesthetic care at our tertiary care center. The secondary objective was to evaluate the common reasons for dissatisfaction and associated expectations.

## METHODOLOGY

This study was conducted for a period of seven months from 1^st^ January 2023 to 31^st^ July 2023 at the surgical wards of our tertiary care hospital. The study subjects included all adult patients planned for non-cardiac elective surgical procedures. All these patients required anaesthesia and overnight hospital admission. While patients having cognitive or behavioral abnormalities, history of depression, or any psychiatric illness were excluded. Patients requiring neurosurgical procedures or a planned postoperative intensive care stay were also excluded. Sample size was calculated based on the study by Alsaif A et al.^1^ In their multicenter study, they reported the overall satisfaction of patients with anaesthetic care was 56.5%. As per reported statistics, we planned to recruit 378 patients to estimate the expected satisfaction rate within a 5% margin of error and 95% confidence interval.

### Ethical Approval:

This cross-sectional survey was approved by the Ethical Review Committee (ERC No: 6774) on 7^th^ December 2022. Written informed consents were obtained from the participants.

The sampling technique was non-probability consecutive sampling. Patients fulfilling the inclusion criteria were followed by one of the primary investigators on their first postoperative day. The survey questionnaire which was available in both English and Urdu was given to all study participants. After reading and understanding the document, they completed it accordingly. The survey form was composed to cover questions regarding the patient’s demographics, satisfaction related to their preoperative (SA), intraoperative (SB), and postoperative care (SC). All these responses were recorded on a 5-point Likert scale including “Very Unsatisfied”, “Unsatisfied”, “Slightly Satisfied”, “Satisfied”, “Very Satisfied”. This corresponds to numerical scoring of one to five respectively.^1^ The factors responsible for dissatisfaction were also assessed by the patient’s response to a particular question.

The results were based on patient responses to individual questions in each section which are shown in percentages. Each section’s percentage was calculated separately. If the percentage of any section was above 50%, it was considered as “Satisfied” from services and if, it was 50% and below, then it was considered as “Unsatisfied”. Each section’s responses were defined separately to determine whether the overall response of any section fell in the “Satisfied” group or the “Unsatisfied” group. For making results, all patients’ responses of “Very Unsatisfied” and “Unsatisfied” which had score of one and two respectively, were considered to fall in the “Unsatisfied” group and patients’ responses of “Slightly Satisfied”, “Satisfied” and “Very Satisfied” which had scores three, four and five respectively were considered to fall in “Satisfied” group.

The questions which were not attempted by the patients were excluded from the response rate of patients and were not included in the results. The data were recorded and analyzed by using the Statistical Package for Social Sciences (SPSS) version 19 (Chicago, Illinois, USA). Demographic information included gender, type of surgery, anaesthesia method, and American Society of Anaesthesiologist (ASA) status. To assess the normal distribution of numerical variables like age, we employed the Shapiro–Wilk or the Kolmogorov-Smirnov test. For numerical data, we computed the mean ± SD or median ± IQR, depending on whether the data followed a normal or non-normal distribution, respectively. Categorical data, such as patients’ satisfaction during different phases of perioperative period and overall satisfaction (SO) was analyzed by calculating frequencies and percentages.

## RESULTS

All enrolled patients filled up the survey with a response rate of 100%. The results were divided into four sections which include:

- Satisfaction regarding preoperative care (SA).
- Satisfaction regarding intraoperative care (SB).
- Satisfaction regarding postoperative care (SC).
- Overall satisfaction (SO).

**Fig.1:**
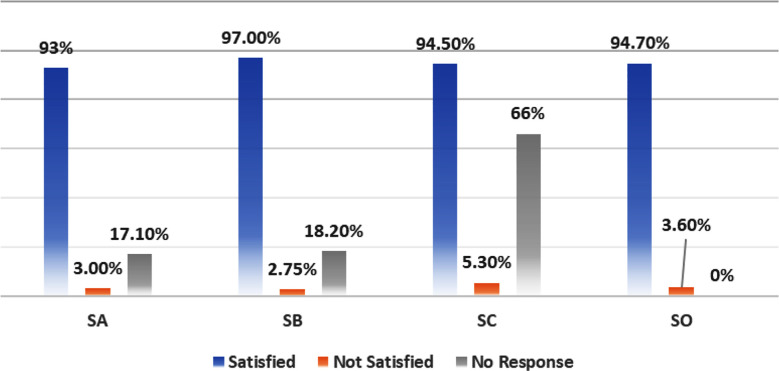
Patients Satisfaction at Perioperative Intervals. SA: Satisfaction related to preoperative care. SB: Satisfaction related to intraoperative care. SC: Satisfaction related to postoperative care. SO: overall satisfaction.

Table-I represents the data related to demographic variables of our participants. The majority of them (67.7%) were female with ASA status II (62.4%). The mean age of patients was 40.6 years. Of these, 33% underwent general surgical procedures. Most of them (95.5%) had General Anaesthesia (GA). Patient satisfaction related to anaesthesia at various time points is shown in Fig.1.

**Table-I:** Demographic Variables.

Variables	Frequency (%) N = 378	P- Value
***Age (Years)***		1.0
Mean (SD)	40.6 (11.5)	
***Gender***		0.85
Female	67.7%	
Male	32.3%	
***Surgical Procedures***		0.92
General Surgery	32.8%	
Obstetrics/Gynecology	26.2%	
Neurosurgery	7.9%	
ENT	5.8%	
Urology	5.8%	
Others	25.2%	
***Type of Anaesthesia***		1.0
GA	95.5%	
RA	4.5%	
***ASA Status***		0.546
I	24.3%	
II	62.4%	
III	13.2%	
***Preoperative Assessment***		0.877
Clinic	85.4%	
Ward	14.6%	

### Regarding Preoperative Care (SA):

More than half of the patients (85.4%) had their assessment done at preoperative clinic. In contrast only 17.7 % patients were evaluated in wards. Comparison of satisfaction showed no significant difference (97.8% vs 95.5%). More than 90% reported that sufficient information had been provided to them regarding anaesthesia. The overall satisfaction of patients regarding their preoperative assessment and related processes was 92.7%. The two major factors that contributed to the dissatisfaction were counselling of patients regarding informed consent (6.4%) and explanation of possible risks and complications related to anaesthetic care (10.4%). The overall dissatisfaction rate was only 3%.

### Regarding anesthetic care (SB):

92.5% of patients interacted with their anaesthetist in the preoperative area, where professionalism was maintained, privacy concerns were addressed, and relevant anaesthetic queries were answered. In 7.4% of patients’ anaesthesiologist was not able to meet them on the day of surgery. In 7.5% of patients’ procedural risks related to regional anaesthesia (RA) and associated complications were not discussed with them. However, overall satisfaction of patients regarding their intraoperative anaesthetic care was 97%, while dissatisfaction rate was only 2.75%.

### Regarding postoperative care (SC):

**Fig.2:**
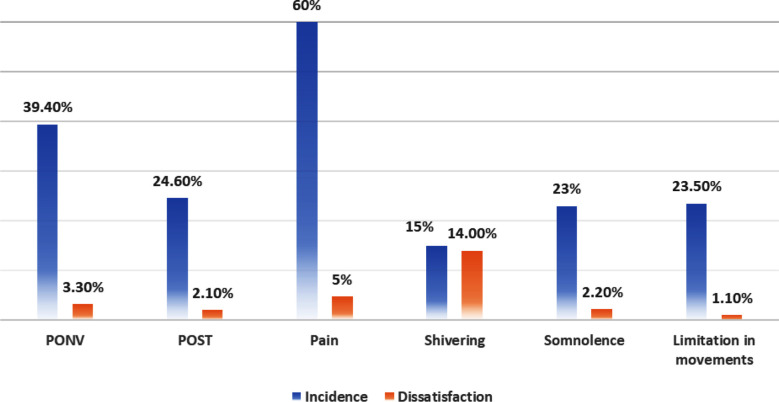
Incidence of common postoperative side effects and dissatisfaction. PONV: Postoperative Nausea and Vomiting. POST: Postoperative Sore Throat.

Around 70% of the participants required anaesthesiologist interaction for associated complaints in the post-anaesthesia care unit (PACU). Amongst them, 96.6% were satisfied. The commonly reported adverse events in recovery were Pain (60%), Postoperative Nausea and Vomiting (PONV) (39.4%), Post-Operative Sore Throat (POST) (24.6%), Somnolence (23%), Limitation in movement (23.5%) and Shivering (15.1%). Amongst them, shivering was associated with the greatest dissatisfaction of around 14%. Fig.2. The overall satisfaction of patients regarding their stay at recovery room was 94.5%, with dissatisfaction of 5.3% which was higher but not statistically significant from SA and SB.

### Overall satisfaction (SO):

When assessing overall satisfaction from participants at intervals A, B and C, 99.5% of patients were satisfied and 0.5% of patients were dissatisfied with the anaesthesia services.

Overall, the p-values across all variables indicate no statistically significant differences in patient satisfaction based on gender, type of surgery, type of anaesthesia, age, or ASA status. This suggests that patient satisfaction in this sample is not strongly associated with any of these demographic or clinical factors as shown in Table-II.

**Table-II:** Comparison of Gender, Type of surgery, Type of anaesthesia, age, ASA status with Satisfaction and Dissatisfaction.

Variables	Satisfied	Unsatisfied	Overall	P-value
	(N=376)	(N=2)	(N=378)	
***Gender***				0.825
Female	254 (67.6%)	2 (100%)	256 (67.7%)	
Male	122 (32.4%)	0 (0%)	122 (32.3%)	
***Surgery***				0.920
ENT	22 (5.9%)	0 (0%)	22 (5.8%)	
General Surgery	123 (32.7%)	1 (50.0%)	124 (32.8%)	
Neurosurgery	30 (8.0%)	0 (0%)	30 (7.9%)	
Obstetrics/Gynaecology	98 (26.1%)	1 (50.0%)	99 (26.2%)	
Urology	22 (5.9%)	0 (0%)	22 (5.8%)	
Others	81 (21.5%)	0 (0%)	81 (21.4%)	
***Type of Anaesthesia***				1.000
GA	359 (95.5%)	2 (100%)	361 (95.5%)	
Regional	17 (4.5%)	0 (0%)	17 (4.5%)	
***Age (years)***				1.000
<40	186 (49.5%)	1 (50.0%)	187 (49.5%)	
>40	190 (50.5%)	1 (50.0%)	191 (50.5%)	
***ASA Status***				0.546
I	92 (24.5%)	0 (0%)	92 (24.3%)	
II	234 (62.2%)	2 (100%)	236 (62.4%)	
III	50 (13.3%)	0 (0%)	50 (13.2%)	

GA: General Anaesthesia. ASA: American Society of Anaesthesiologist of Physical Status.

## DISCUSSION

This study aimed to assess patient satisfaction regarding anaesthetic care in a tertiary care center in Pakistan, focusing on preoperative, intraoperative, and postoperative period. The findings indicate high satisfaction rates across all phases of care. This reflects quality of anaesthesia services provided. The overall score was 94.7%, with notable satisfaction in the intraoperative and postoperative phases, standing at 97% and 94.5% respectively. The key findings of our study align with previous research highlighting patients’ satisfaction as a critical aspect of quality care.^10^ Moreover, association between preoperative counselling and effective postoperative pain management is also well established. Despite the high satisfaction rates, factors contributing to dissatisfaction needs to be critically examined. Notably, 10.4% of our patients reported insufficient counseling about risks and complications related to anaesthesia. Along with routine preoperative assessment, anaesthetist should also focus on emotional aspects related to surgery and when relevant, evaluate previous experiences. The discussion should also include explanation about common risks and discomforts associated with perioperative care.^11^ To us, it is equally important to know the common reasons for dissatisfaction, no matter how small number of patients had experienced that.

A large retrospective study done in Japanese hospital^12^ showed dissatisfaction rate of 5.82%. Preoperative medical conditions, association with RA and PONV were the key factors responsible for that. The reported dissatisfaction of our participants was highest (5.3%) in postoperative phase (SC). Highlighted causes here were Pain, PONV, POST, Shivering, limitation of movements and somnolence. One of our previous studies conducted on neurosurgical patients at PACU^13^ showed Pain, POST, PONV and shivering as commonly reported events. However, no specific association was linked with age, gender and type of surgical procedure. Nonetheless, consistency in trends is important for implementing quality initiatives. The issue of postoperative pain is complex requiring focused discussion on risks, complications, and overall benefit of used regime. The reported incidence in advanced health care setups remains significantly high. The recent large cross-sectional study includes data of around 26,193 postoperative patients revealed incidence of around 48.7%.^14^

The study showed a clear association with satisfaction and reinforced the need of patient involvement in terms of shared decision making. Historically, the PONV is associated with significant dissatisfaction related to anaesthesia. This is mainly because of associated discomfort, complication, increasing length of stay and hence the cost.^15^ Competent anaesthesia teams should focus on risk prediction, modification of anaesthesia techniques and evidence-based antiemetic prophylaxis. The other significant factor contributing to dissatisfaction in our study was postoperative shivering. Around 14% of patients reported it as distressing. This finding is consistent with recent data where the reported frequency of shivering was 50.6%.^16^ The common risk factors associated with shivering are hypothermia, hypotension and prolonged surgical time. Recommended strategies include using active warming devices and limiting the associated risk factors.^17^

Overall, the result of our study highlights the strength of following perioperative standards, the prime importance amongst which is routine preoperative assessment, risk stratification and getting informed consent. The overall satisfaction is also linked to the type of administrative setup we are working, and what standards we are following. Even with the implementation of mentioned strategies, the issue is complex, dependent on past experiences, various socioeconomic and cultural factors and educational background.^18^

### Limitations:

The prime one is single center outcomes, and the results cannot be generalized considering wide variation in administrative setups, clinical practices and patient demographics across the country. However, the results would be very useful in contributing to the concept at a national forum and measuring tools can easily be translated to any kind of health care setup. Moreover, well conducted randomized controlled trial would be the better way to judge satisfaction considering various demographic variations and also the type of anaesthesia. These trials should also give special consideration to previous exposure and past experiences related to anaesthetic care.

## CONCLUSION

While most of the patients reported satisfaction with their anaesthesia care, addressing gaps in informed consent, explaining associated risks, and preventing postoperative discomforts such as Pain, POST and shivering could further enhance patient satisfaction. The role of the anaesthesiologist needs to be established in preoperative assessment and shared decision making.

### Authors’ Contributions:

**UAB:** Concept and design of the study, literature search, Conceived the study, Acquisition of data and analysis, Interpretation of Data

**FS:** Revision for critically important intellectual content, Reviewed the manuscript, final approval of the version to be published, monitored the whole study, and agreed to be accountable for all aspects of work.
